# Multiple Cerebral Hydatid Cysts: A Case Report

**DOI:** 10.7759/cureus.25529

**Published:** 2022-05-31

**Authors:** Yassine Akrim, Knza Barkate, Yassine Arrad, Houssine Ghannane, Awatif El Hakkouni

**Affiliations:** 1 Biology Department, Parasitology and Mycology Laboratory, Mohammed VI University Hospital of Marrakech, Faculty of Medicine and Pharmacy, Marrakech Cadi Ayyad University, Marrakesh, MAR; 2 Neurosurgery Department, Mohammed VI University Hospital of Marrakech, Faculty of Medicine and Pharmacy, Marrakech Cadi Ayyad University, Marrakesh, MAR

**Keywords:** hydatid cyst, cerebral hydatid cysts, cerebral hydatidosis, parasitic disease, zoonotic infection, echinococcus granulosus, echinococcosis, hydatidosis

## Abstract

Hydatidosis is an endemic disease in Morocco. Intracranial localization is relatively unaccustomed and usually occurs in a solitary form. It is even rarer to find multiple intracranial hydatid cysts. Patients are frequently children and young adults. We report the case of a 22-year-old woman with recurrent multiple cerebral hydatid disease occurring seven years after resection of a primary cyst. She was admitted due to high intracranial pressure and generalized seizures. Brain CT scan showed several intracranial multivesicular cysts in the left parieto-occipital region with localized calcifications. According to the radiological results and patient story, the diagnosis of cerebral hydatidosis was presumed. The patient underwent complete excision of the cysts followed by medical therapy. The parasitological and histological examination of the surgical specimen confirmed the diagnosis. The transient neurological deficit was the only postoperative complication improved, thanks to reeducation in the early phase. The patient was discharged in good condition with no other complications at the follow-up.

## Introduction

Human echinococcosis is a zoonotic parasitic disease caused by *Echinococcus granulosus*, which forms larval cysts in the human tissue. *Echinococcus granulosus* is a tapeworm that lives in the intestines of dogs that are the definitive hosts. Infected dogs pass tapeworm eggs in their stool, which can be consumed by sheep, cattle, goats, pigs, and accidentally humans as intermediate hosts. Hydatidosis is a public health problem particularly in the endemic areas represented by sheep-raising countries, including Morocco [1].

The liver is the most common organ affected, followed by the lungs. Intracranial hydatid disease is a rare presentation seen in children and young adults, with reported incidence of 2% of all cases [2]. It might be primary or secondary. Multiple cerebral hydatid cysts in the brain are uncommon [3].

We describe the case of a 22-year-old woman with recurrent multiple cerebral hydatid disease occurring seven years after resection of a primary cyst.

## Case presentation

A 22-year-old female patient from a rural area was admitted to our hospital with features of raised intracranial pressure: headache, blurred vision, and vomiting. She also reported generalized tonic-clonic seizures over the previous years. In her medical history, she stated that she had an operation to remove a cerebral cyst at another surgical center. When asked for more details, history of an intracranial solitary cyst ruptured during excision was reported seven years back, which was diagnosed to be hydatid cyst after biopsy and serological investigations. The diagnosis of recurrent cerebral hydatidosis with multiple cysts was suggested. During admission time, examination revealed a afebrile and conscious patient, hemodynamically stable, the pulse rate, blood pressure, and oxygen levels were normal. Clinical examination findings were unremarkable, and no abnormality in the neurological examination was noted. Blood investigation revealed the following results: raised total leukocyte count of 12,900 cell/mm^3^, hemoglobin of 13.8 g/dL, platelets of 239,000 cells/mm^3^, and C-reactive protein (CRP) of 3 mg/L. The results of the other tests (serum electrolyte levels, liver function, kidney function, and coagulation tests) were normal.

**Figure 1:**
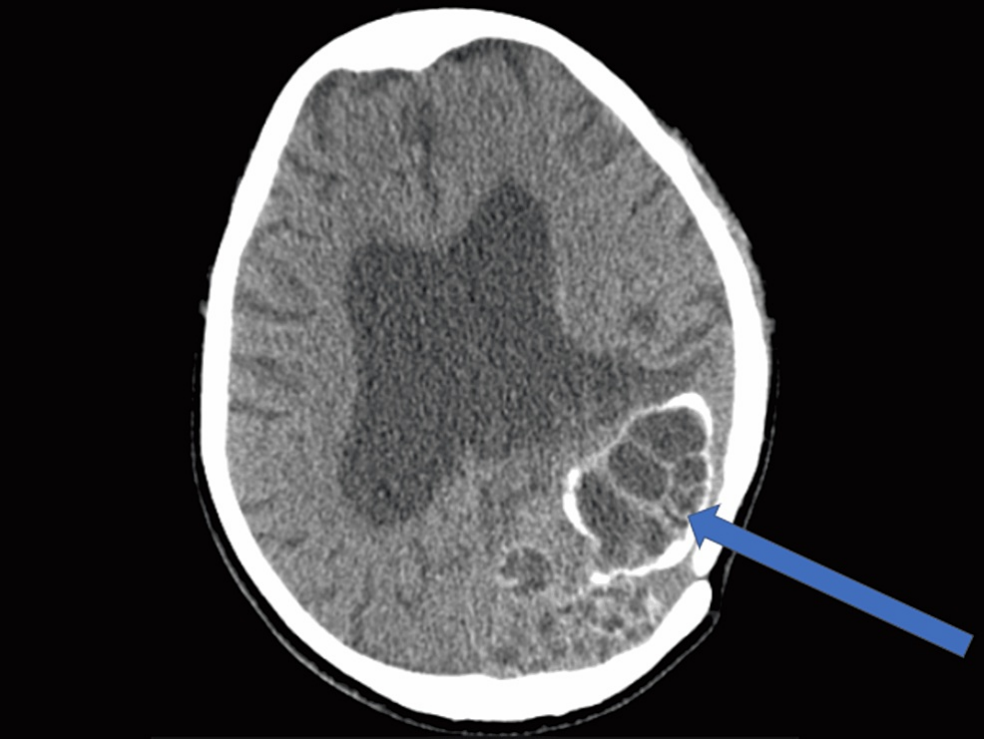
CT scan showing axial image of multiple intracranial multivesicular cysts in the left parieto-occipital region with localized calcifications (arrow).

A computed tomography (CT) scan of the head showed hydrocephalus with multiple intracranial hydatid cysts in the left parieto-occipital region (Figure 1). Based on the cranial imaging findings and the patient history, the diagnosis of recurrent cerebral hydatid disease with multiple cysts of the brain was suggested.

Upon surgical exploration, using the technique of hydrodissection, as described by Arana-Iniguez and San Julian, the cysts were totally extracted without rupture. The surgical areas were cleaned with sodium chloride. The removed material was sent to the Parasitology Laboratory for analysis.

**Figure 2:**
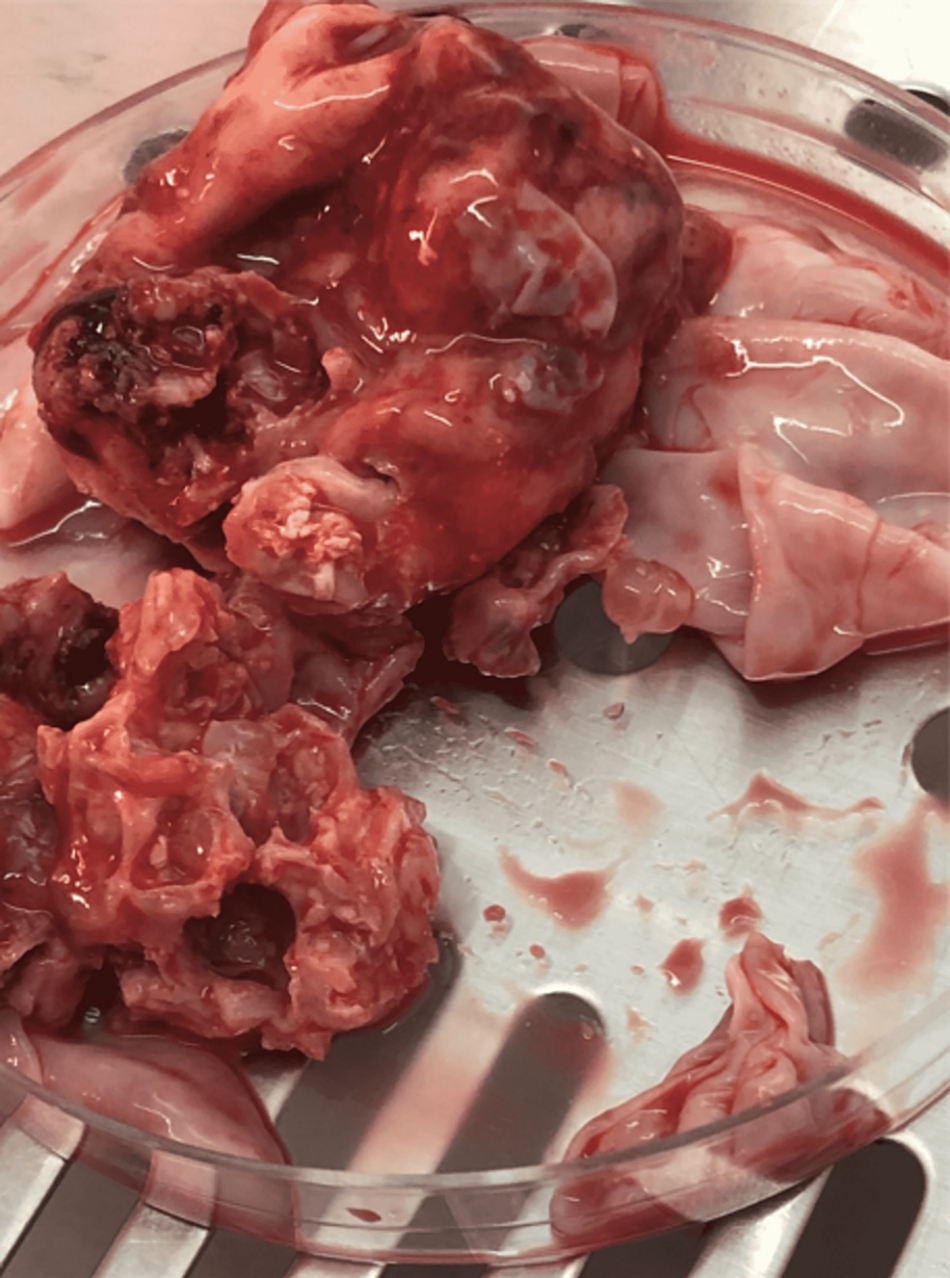
Macroscopic findings of extracted hydatid cysts. Whitish cysts filled by multiple variable-sized daughter cysts.

The macroscopic analysis of the surgical specimen revealed spherical cysts with whitish walls. Several pearly white translucent daughter cysts of variable sizes, with clear fluid and membranous structures, were observed inside the cysts (Figure 2).

**Figure 3:**
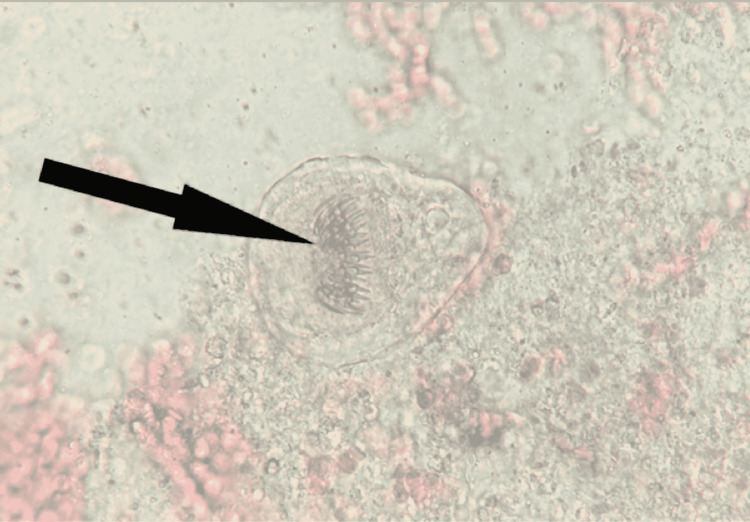
Direct microscopic examination (40x magnification) showing protoscoleces. Note the row of hooklets (arrow).

**Figure 4:**
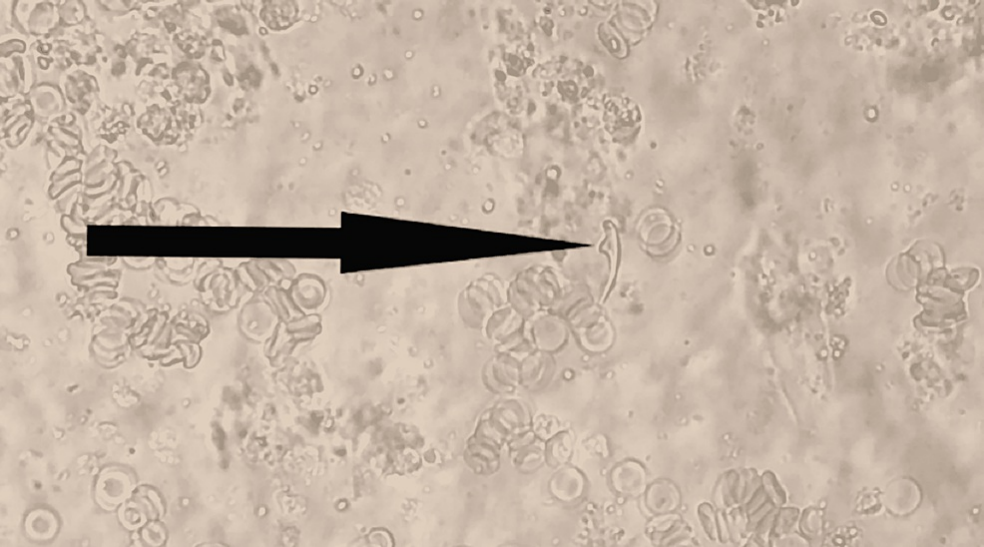
Free hooklet from the hydatid cyst fluid observed by microscopy (40x magnification).

After centrifugation of the hydatid cyst fluid, microscopic examination showed several invaginated protoscoleces with row of hooklets (Figure 3) and also free hooklets of Echinococcus (Figure 4).

Histopathologic examination of the specimen confirmed the diagnosis of hydatid cyst by visualizing the laminated membrane of the cyst wall.

Our patient was treated with albendazole under supervision of an infectious disease specialist. The patient stayed in the intensive care unit for two days after the operation and was then transferred to the Neurosurgery Department. As a post-operative complication, our patient developed transient neurological deficit, which gradually recovered with early rehabilitation. She showed marked recovery in her neurological status. Therefore, she was discharged with long-term prescription of oral albendazole at 400 mg/day given in cycles of 28 days interrupted by a 15-day period with a routine monitoring of blood counts and liver function tests (in view of the potential hepatic and hematological adverse effects of albendazole).

## Discussion

Hydatidosis is currently considered as an endemic zoonotic disease in Morocco. A total of 23,512 human cases were noticed by the Ministry of Health in Morocco during the periods 1980-1992 and 2003-2008 [1]. Hydatid disease occurs by infection with the larval stage of the tapeworm *Echinococcus granulosus*. Humans become infected by ingestion of eggs from the dog hydatid tapeworm due to poor hygiene practices or contaminated food and water [4].

Hydatid disease is usually observed in the liver (60%) and lungs (30%). However, any organ may be involved. Cerebral hydatid disease is a rare entity [5]. It is more common in children and young adults (50 to 70% of cases) [6]. This parasite passes through the hepatic and pulmonary filter and reaches the brain through the systemic circulation. The growth of hydatid cysts is slow. The disease usually goes undetected until cysts are large enough to produce symptoms [6]. Brain hydatidosis can be primary or secondary. The direct invasion of the brain by the larvae, without affecting other tissues, represents the primary form. In our case, cysts contain brood capsules released from the germinal membrane and protoscolices. Primary cysts are characterized by high fertility, and their rupture may be responsible for recurrent forms. Secondary cysts result from a fertile primary cyst, which has ruptured following trauma or during surgery or spontaneously. These cysts lack germinal membranes and are infertile. In case of their ruptures, the recurrence is exceptional [7]. The clinical presentation depends on the area of the brain affected. Cephalalgia, motor weakness, vomiting, nausea, symptoms of cranial nerve disorders, and seizure are the typical clinical manifestations [8]. Our patient presented with raised intracranial pressure syndrome with vomiting and headache.

MRI and CT scan help in the diagnosis and appropriate treatment. They often detect a round well-margined cystic lesion without marginal enhancement, although there are no pathognomonic imaging findings [9]. Our radiological findings in this report showed multiple intracranial hydatid cysts in the left parieto-occipital region. Differential diagnosis includes other cystic lesions such as arachnoid and porencephalic cysts, cystic tumors, and cerebral abscess [10].

Concerning biological tests, the Casoni skin test is not sufficiently reliable since it is only positive in 60% of cases in cerebral hydatidosis [11]. Currently, the tests used include sensitive screening tests, such as ELISA (enzyme-linked immunoassay), and specific confirmation tests, such as Western blotting or equivalent procedures. The diagnostic sensitivity of ELISA tests is between 60% and 90% [12]. Due to cross-reactions, we may have false-positive results from patients with another parasitic disease. We can also have false-negative results due to a minimal immune response induced within the brain by intracranial hydatid cyst [13]. Hydatid disease can be diagnosed by serology and imaging studies, but these are not definitive. A confirmative diagnosis of hydatid disease can only be made by microscopic demonstration of the parasite [14].

The treatment of hydatid cyst is exclusively surgical. It is paramount to remove the cyst without rupture in order to avoid complications such as spread of parasites, recurrence, and anaphylactic reaction [15]. The most common surgical approach is that described by Arana-Iniguez and San Julian and consists of forced expulsion of the cyst by hydrodissection introducing hypertonic saline solution under and around the cyst [16].

Cerebral hydatidosis is considered by most studies as a benign disease. Nevertheless, it can cause neurologic sequelae and postoperative complications such as seizure, subdural hematoma, bacterial meningitis, porencephalic cyst, and hydrocephalus [6,17]. Medical treatment with albendazole is generally used to treat intraoperative ruptured cysts, recurrences, disseminated, and inoperable forms [6].

## Conclusions

Multiple intracerebral hydatid cysts are a rare condition even in endemic regions such as Morocco. Cerebral hydatid disease should be suspected particularly when cystic lesions appear in the central nervous system. The clinical features are those of intracranial space-occupying lesions. Radiological imaging modalities, especially MRI and CT scan, are mandatory for accurate diagnosis. This disease can be managed by surgical treatment for a complete excision of the cyst without rupture. Preventive measures are the only way to control the hydatid disease and eventually eradicate it.
